# Simultaneous editing of two *DMR6* genes in grapevine results in reduced susceptibility to downy mildew

**DOI:** 10.3389/fpls.2023.1242240

**Published:** 2023-08-21

**Authors:** Lisa Giacomelli, Tieme Zeilmaker, Oscar Giovannini, Umberto Salvagnin, Domenico Masuero, Pietro Franceschi, Urska Vrhovsek, Simone Scintilla, Jeroen Rouppe van der Voort, Claudio Moser

**Affiliations:** ^1^ Research and Innovation Centre, Fondazione Edmund Mach, San Michele all’Adige, Italy; ^2^ Enza Zaden Research & Development B.V., Enkhuizen, Netherlands

**Keywords:** gene editing, DMR6, *Vitis vinifera*, downy mildew, susceptibility gene, *Plasmopara viticola*, salicylic acid

## Abstract

The reduction of pesticide treatments is of paramount importance for the sustainability of viticulture, and it can be achieved through a combination of strategies, including the cultivation of vines (*Vitis vinifera*) that are resistant or tolerant to diseases such as downy mildew (DM). In many crops, the knock-out of *Downy Mildew Resistant 6* (*DMR6*) proved successful in controlling DM-resistance, but the effect of mutations in *DMR6* genes is not yet known in grapevine.

Today, gene editing serves crop improvement with small and specific mutations while maintaining the genetic background of commercially important clones. Moreover, recent technological advances allowed to produce non-transgenic grapevine clones by regeneration of protoplasts edited with the CRISPR/Cas9 ribonucleoprotein. This approach may revolutionize the production of new grapevine varieties and clones, but it requires knowledge about the targets and the impact of editing on plant phenotype and fitness in different cultivars.

In this work we generated single and double knock-out mutants by editing *DMR6* susceptibility (S) genes using CRISPR/Cas9, and showed that only the combined mutations in *VviDMR6-1* and *VviDMR6-2* are effective in reducing susceptibility to DM in two table-grape cultivars by increasing the levels of endogenous salicylic acid. Therefore, editing both genes may be necessary for effective DM control in real-world agricultural settings, which could potentially lead to unwanted phenotypes. Additional research, including trials conducted in experimental vineyards, is required to gain a deeper understanding of *DMR6*-based resistance.

## Introduction

Grapevine (*Vitis vinifera L*.) is a widely cultivated crop of great economic value; the global area dedicated to viticulture is 7.4 Mha (2019 estimate) to produce table grapes, wines, juices and raisins (Bois et al., 2017). In humid weather, the most devastating disease affecting grapevine is downy mildew (DM), caused by the oomycete *Plasmopara viticola*.

The use of pesticides as a preventive measure against DM is becoming increasingly unsustainable in terms of cost, human health, and environmental concerns. To substantially reduce chemical treatments, one approach is to breed resistant plants that possess a wide range of resistances. This can be achieved by introducing resistance (R) loci from *Vitis* spp. other than *V. vinifera* (reviewed by Vezzulli et al. (2022)). Some *Rpv* (*Resistance to Plasmopara viticola*) loci have been identified at the gene level (Feechan et al., 2013; Foria et al., 2020). However, the effectiveness of R loci resistance is typically limited to specific pathogens and can be overcome within a few years after their introgression due to pathogen evolution. Additionally, the success of new DM-resistant varieties is hindered by the wine-making industry’s adherence to traditional cultivars. Another approach to achieving DM-resistance in grapevine is through gene editing, which allows for precise and targeted mutations. Today, this technique holds the potential to generate resistant clones of traditional varieties in a DNA-free manner (Najafi et al., 2022; Scintilla et al., 2022). Moreover, by engineering susceptibility (S) genes such as *Downy mildew resistant 6* (*DMR6*) it becomes feasible to achieve broad-spectrum resistance. Mutations in DMR6 genes provide resistance to oomycetes as well as fungi and bacteria in several horticultural crops as well as tree species including banana, apple, and citrus (de Toledo Thomazella et al., 2021; Hasley et al., 2021; Kieu et al., 2021; Shan et al., 2021; Tripathi et al., 2021; Parajuli et al., 2022).

The susceptibility gene *DMR6* encodes a 2-oxoglutarate Fe(II)-oxygenase and functions as negative regulator of immunity. Together with DLO (DMR6-like oxygenase) it was shown to function as a salicylic acid (SA)-hydroxylase (Zhang et al., 2013; Zhang et al., 2017). DMR6 and DLO convert SA to 2,5- and 2,3- dihydroxybenzoic acid (DHBA), respectively, and are key-players in maintaining SA homeostasis during plant growth and response to (hemi-) biotrophic pathogens.

In grapevine, the *DMR6-DLO* family consists of two highly similar *DMR6* (*VviDMR6-1* and *VviDMR6-2*) and three *DLO* genes (*VviDLO1*, *2* and *3*). A recent network analysis of transcriptionally co-regulated genes revealed that a consistent group of defense-associated genes are co-regulated especially with *VviDMR6-1*, and that over-expression of *VviDMR6-1* restores DM-susceptibility in the Arabidopsis *dmr6-1* resistant mutant (Pirrello et al., 2022). Collectively, these data indicate *VviDMR6-1* as a favorite candidate to be engineered for DM-resistance, but the contribution of each individual gene of the family remains to be investigated, as well as the effect of mutations on the plant phenotype in different cultivars. SA is also involved in regulating growth and development, and its accumulation tightly regulated in a speciesdependent manner (Van Butselaar, 2023). It is therefore key to understand how SA is regulated in grapevine to exploit *DMR6* as a target gene for resistance to biotrophic pathogens. In this work, we generated single and double mutants in grapevine by editing *VviDMR6-1* and *VviDMR6-2* using CRISPR/Cas9, and showed that combined mutations in *VviDMR6-1* and *VviDMR6-2* are required to reduce susceptibility to DM in two table-grape cultivars by increasing the levels of endogenous SA.

## Material and methods

### CRISPR/Cas9 constructs

Guide RNAs to specifically target grapevine *DMR6* genes were designed on the Pinot PN4004 reference genome using the CRISPR-P software tool (Lei et al., 2014), cloned into level 1 expression vectors behind the AtU6 promoter, and subsequently into a binary vector (pAGM4723) containing a domesticated Cas9 driven by a double 35S promoter (Nekrasov et al., 2013; Hahn et al., 2020). Backbone vectors for Golden Gate cloning were obtained from the Addgene plasmid repository (www.addgene.org, plasmids 46968, 51144, 49771, 48002, 48003, 48018, 48019, and 48015). Target and PAM site regions were sequenced by Sanger and Next Generation Sequencing (NGS) in the different cultivars to check for absence of polymorphisms. The sgRNAs used in this work were: GCCGATGCTTGCAGGCTCTA (DM1a) and GTCCTTGCCGAGGTCGATTA (DM1b) for the *VviDMR6-1* gene (Vitvi16g01336); GGGCTCGATCGTCACAACTC (DM2a), GATGTAGTTCTCCGGCAAAG (DM2b), and GGAGGATTGGAGGGCCACTC (DM2c) for the *VviDMR6-2* gene (Vitvi13g01119). Guides DM1a and DM1b were used in the constructs pDM2a and pDM2b, respectively, pDM1a and pDM1b to edit *VviDMR6-1* alone; guides DM2a and DM2b were used in the constructs pDM2a and pDM2b, respectively, to edit *VviDMR6-2* alone. In addition, vectors for simultaneous expression of two sgRNAs were constructed: pDM1a2a (with guides DM1a and DM2a), pDM1a2b (with DM1a and DM2b), and pDM1a2c (with DM1a and DM2c) to edit simultaneously *VviDMR6-1* and *VviDMR6-2* (**Table S1**).

### Detection of off-target editing

Possible off-target sites were detected by BLAST search for near matches against the PN40024.v4 assembly in EnsemblPlants (http://plants.ensembl.org) using the sgRNA target sequences as query. *dmr6-1* and *dmr6-2* mutants included in later phenotypic analyses were sequenced to detect possible off-target mutations in regions with a mismatch of up to two nucleotides with the guide RNAs. Off-target mutations were analyzed by Sanger sequencing and NGS.

### Plant material and gene transfer

Embryogenic calli were obtained from cultures of ovaries and anthers of *V. vinifera* cv. Chardonnay, Merlot, Sugraone, Crimson seedless, Thompson seedless, and microvine 04C023V0006 (Chaïb et al., 2010) according to Martinelli et al. (2001). These calli were cultivated in absence of selection to regenerate the wild type plants used in this work, and in parallel they were transformed by co-cultivation with *Agrobacterium tumefaciens* EHA105 carrying the proper binary vector, essentially as described by Dalla Costa et al. (2016). Embryogenesis was induced in the dark on NN solid medium (Nitsch and Nitsch, 1969) supplemented with 1 g/L activated charcoal, 45 g/L sucrose, 150 *µ*g/L kanamycin, 1 mg/L timentin, 0.9 *µ*M 6-benzylaminopurine, 11.4 *µ*M indole-3-acetic acid, and 10 *µ*M beta-naphthoxyacetic acid. Depending on the cultivar, embryos were cultivated on NN solid medium, 15 g/L sucrose, and 25 *µ*g/L kanamycin with or without the supplement of hormones (4.5 *µ*M 6-benzylaminopurine and 5 *µ*M indole-3-butyric acid) in the light (16 h photoperiod). Transgenic lines were screened by PCR with NPTii specific primers (GCCAACGCTATGTCCTGATA, and ACAATCGGCTGCTCTGATG). DNA was extracted from edited lines and analyzed by targeted amplicon NGS sequencing: short amplicons containing 5-overhang adapters were generated to make the Illumina libraries (Illumina, San Diego, CA, USA) and reads were analyzed through the CRISPResso platform (Pinello et al., 2016) using standard settings for filtering of low-quality reads and trimming of adapter sequences. In addition, the transgene free *dmr6-2* mutants (lines H1D, H1C, H1C1) were obtained via single-cell technology by editing of Crimson seedless protoplasts with the ribonucleoprotein complex: TrueCut Cas9 Protein v2 (Thermo Fisher Scientific, Waltham, MA, USA) and sgRNA DM2c, as described in Scintilla et al. (2022).

### Plant growth

Regenerated plants (both wild type and edited) were propagated on NN medium with 15 g/L sucrose and maintained in a climate-controlled chamber: 16 h light photo-period, 23°C, 60% relative humidity (RH). Plants were acclimatized in rooting soil with low percentage of pumice, and grown in a clean environment under LED light (400 *µ*mol photons m^−2^ s^−1^ at the plant-tip height, 16 h light photo-period), and then transferred to a greenhouse under natural light conditions. Plants were regularly watered.

### DM-assay on leaf discs

*P. viticola* (Berk. & M.A. Curtis) Berl. & De Toni was propagated on susceptible plants in the greenhouse. Symptomatic plants were placed in the dark at 100% RH overnight to induce sporulation. The inoculum was prepared by suspending sporangia in cold water. Discs were obtained from leaves of untreated and healthy plants, maintained in a growth-chamber (16 h light photo-period, 23°C, 60% RH). Leaf discs (0.8 cm diameter) were cut from the third to ninth leaf of actively growing stems, and laid–abaxial-side up–on four sheets of wet absorbing-paper in Microbox containers (Sac O2, Nevele, Belgium) in the light at 25°C. To account for ontogenic resistance, leaves were grouped according to their age, as determined by their position on the stem. Leaves of the same age group were treated together and compared within the group. Discs were sprayed with suspension of 10^5^ sporangia/mL. Severity—calculated as percentage of the leaf-disc area showing sporulation—and incidence were evaluated at 7 days post inoculation (dpi) with the ImageJ software. The average severity of the wild type was used as internal control within each experiment, and used to normalize data across experiments. The lines used were: A43, and S93 (*dmr6-1*) M60, and M87 (*dmr6-2*), M57 (*dmr6-1_2*) of cv. Sugraone, and D39 (*dmr6-1*), H1D1 (*dmr6-2*), M54, M42, O26 and O79 (*dmr6-1_2*) of cv. Crimson seedless.

### *In planta* DM-assay

Healthy plants were acclimatized to greenhouse conditions, and pruned to synchronize growth. Plants were grown for up to six weeks, and treated weekly (Cydely top, Syngenta); treatments were suspended 10 days prior to the DM-assay. Plants were sprayed with a suspension of 2.5x10^5^ sporangia/mL with the aid of an air-compressor, and left at 100% humidity overnight. Sporulation was induced by 100% RH at 6 dpi. Severity was calculated as percentage of the leaf area showing sporulation on a 10%-step scale. Severity was measured on leaves of different age, inferred by their position on the stem on actively-growing shoots. Two experiments were performed considering multiple lines per genotype and plants of the same age within each experiment. The following plants were considered in the first experiment: 15 Crimson seedless plants including three *dmr6-1* (lines D39 and D56); one *dmr6-2* (line H1C1), four *dmr6-1_2* (lines M42, M43, M54 and O26), and six wild types. In the Sugraone background the following 15 plants were used: three *dmr6-1* plants (lines S93 and A93), two *dmr6-2* (lines M60, and M87), six *dmr6-1_2* (lines N12 and M57), and four wild types. Due to the elevated number of plants, in the second experiment the two cultivars were treated separately; the following 33 Crimson seedless plants were considered: eight *dmr6-1* (lines E7, B95, D39, and D56), five *dmr6-2* (lines H1C1, and H1D), 14 *dmr6-1_2* (lines M42, M54, O26, O79, and P21), and six wild type plants. In the Sugraone background the following 22 plants were used: seven *dmr6-1* (lines A43, A50, S93 and A93), three *dmr6-2* (lines M60, M86, and M87), seven *dmr6-1_2* (lines N12 and M57), and five wild type plants. Data relative to the first two (youngest) leaves were not considered due to variability in density and development of the stomata, and thickness of the cuticle.

### Statistical analyses

All DM-severity data analyses were performed in R (R Core Team, 2021) relying on the tidyverse package Wickham et al. (2019). In all cases the measurements of severity (bounded between 0 and 1) were subject to logit transformation prior to statistical modeling (Warton and Hui, 2011). In the case of the in planta DM-assay, only leaves of growing shoots were included in the analysis. The first two leaves were always discarded and the remaining were divided into two groups (*<* 9, and ≥ 9) to account for their expected difference in susceptibility (ontogenic resistance). The data measured for each leaf were aggregated at the level of the plant by using a Generalized Linear Model (GLM). The estimates of such a model were then used to compare the three edited genotypes against the wild type. Since the data were collected across different experiments, a substantial batch variability is expected. In order to compensate for that, the average transformed severity of each experimental run—which was containing all four genotypes—was set to zero. In the case of the DM-assay on leaf-discs, severity was assessed on the individual discs due to the limited number of plants included in this preliminary investigation. Also in this case, the data were collected across a set of independent experiments, which did not always include the full set of genotypes. In this case, to remove the batch effect, the average transformed severity of the wild type was set to zero. As for potted plants statistical testing was performed by a GLM. SA and DHBA quantification data were log-transformed and tested with one-way parametric ANOVA in Python within the Google Colab platform.

### SA determination

DHBAs (including SA) were extracted from leaf discs (0.1 g FW) cut from the 10*^th^* and 11*^th^* leaf (actively growing stem) of greenhouse-adapted plants, according to Zeilmaker et al. (2015): free DHBAs were extracted in methanol spiked with 100 ng SA4d (Merk KGaA, Darmstad, Germany). DHBAs were analyzed by LC–MS/MS on an ExionLC system interfaced with AB6500+ QTrap with electrospray ionization system (Applied Biosystems/MDS Sciex, Toronto, ON, Canada). All samples were analyzed on a reversed phase ACQUITY UPLC 1.8 m 2.1 × 150 mm HSS T3 column (Waters, Milford, MA, USA) at 40°C and with a mobile-phase flow-rate of 0.28 mL/min using solvents A (0.1% v/v formic acid in water) and B (0.1% v/v formic acid in methanol) in a linear gradient: from 10% B to 100% B in 18 min; 18-20 min, 100% B isocratic; 20–20.1 min, 100–10% B; 20.1–23 min 10% B isocratic. The injection volume was 2 *µ*L. The transitions and spectrometric parameters were optimized individually for each standard (5 µg/mL). The two most abundant fragments, to be used as the quantifier and qualifier, were identified for each compound. The compound-specific instrumental parameters are shown in **Table S2**. The spray voltage was set at 5500V for positive mode and -4500V in negative mode. The source temperature was set to 500°C, the nebulizer gas and heater gas to 50 and 60 psi, respectively.

## Results and discussion

### Generation of mutants

Mutant classes are referred to as ‘genotypes’ (e.g. *dmr6-1*, *dmr6-2*, *dmr6-1_2*), the individual transformation products within each genotype as ‘lines’, and non-edited plants as ‘wild type’ (non transformed and regenerated from the same callus as the mutant lines).

**Figure 1:**
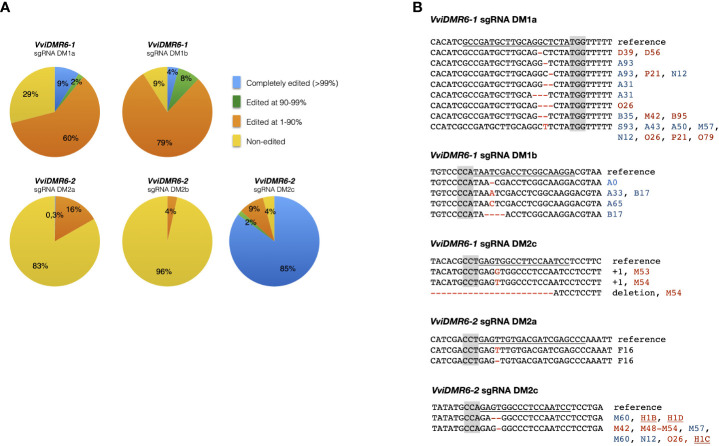
Genotype of selected edited plants. **(A)** Grouping of edited plants based on the fraction of edited reads in NGS sequencing. Completely edited plants were defined as those with more than 99% of edited reads. **(B)** Editing types of plants selected for phenotypic analysis. Color coding: PAM sequence (gray); Sugraone (blue), Crimson seedless (red), Thompson seedless (black). Non-transgenic *dmr6-2* mutants obtained from edited protoplasts are underlined.

Embryogenic calli of six cultivars were transformed, and 994 kanamycin-resistant regenerated plants were sequenced: 236 transformed with pDM1a, 100 with pDM1b, 147 with pDM2a, 198 with pDM2b, 192 with pDM1a2a, 121 with pDM1a2c (**Table S1**). Among all cultivars, the calli of Sugraone and Crimson seedless were the most regenerative, and therefore those producing the highest number of completely edited plants. We analyzed further only Crimson seedless and Sugraone plants because only in these two cultivars we obtained the full set of completely edited single (*dmr6-1*, *dmr6-2*) and double (*dmr6-1_2*) mutants. Considering that (i) even traces of wild type sectors may mask the phenotype of a recessive mutation, and (ii) genetic chimeras are a common product of callus co-cultivation with Agrobacterium, we relied on NGS to select plants with editing in at least 90% of the reads (blue and green sectors in pies of **Figure 1A**). The discarded group (orange) included non-edited and heterozygous plants, as well as chimeras with non-edited portions. Only plants edited in over 99% of the reads (blue sectors of pies in **Figure 1A**) showed a stable genotype in propagated cuttings over time (**Figure S1**), which we would have missed by relying only on Sanger sequencing for selection of mutants. We refer to these lines as ‘completely edited’; the editing types of completely edited lines selected for further phenotypic analysis are illustrated in **Figure 1B**. Complete editing of *VviDMR6-1* was obtained with both DM1a and DM1b sgRNAs. Editing of *VviDMR6-2* with DM2a was effective only in cv. Thompson seedless (**Figure 1B**, **Table S1**), while DM2b was virtually ineffective. Conversely, 85% of the screened plants were completely edited (**Figure 1A**), and mostly mono-allelic when DM2c was used. This guide was used in a ribonucleoprotein complex with Cas9 to edit Crimson seedless protoplasts and to regenerate non-transgenic (DNA-free) *dmr6-2* mutants (Scintilla et al., 2022), namely lines H1C, H1D, and H1C1 (**Figure 1B**).

Mutants included in phenotypic analyses were checked for possible off-target editing: DM2c produced off-target mutations in *VviDMR6-1* in all transgenic *dmr6-2* mutants in Crimson seedless—therefore considered double mutants—but not in Sugraone (lines M60, M61, M86, M87), suggesting that editing efficiency depends both on the guide and on the genetic background (**Figure 1B**).

### Reduced susceptibility to DM of *dmr6* mutants

**Figure 2:**
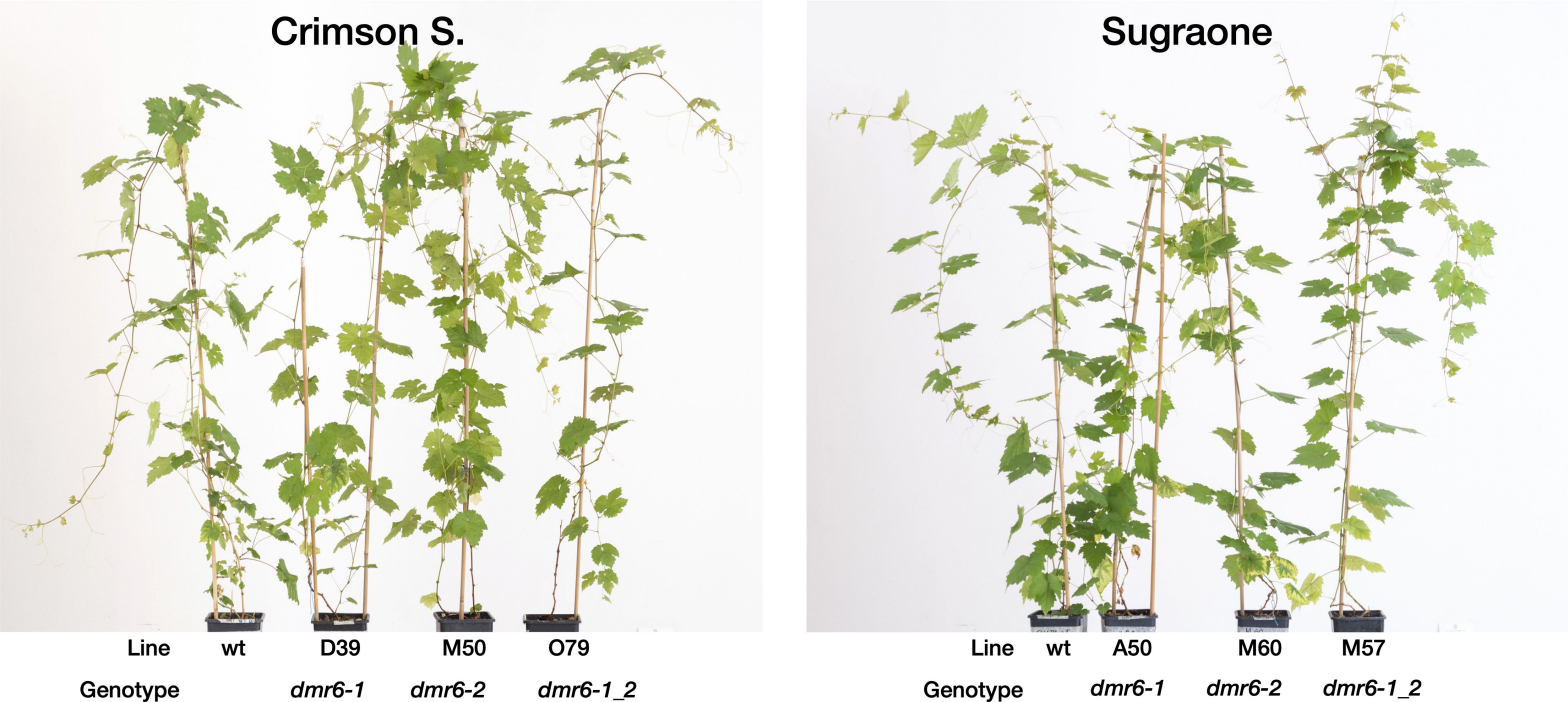
Images of mutants in two genetic backgrounds: Greenhouse-adapted plants of cv. Crimson seedless and Sugraone. From right to left are the non-edited wild type, and lines of *dmr6-1*, *dmr6-2*, and double *dmr6-1_2* mutants. Pictures were taken six weeks after pruning, at the time of DM-inoculation.

Single and double *dmr6* mutants in cultivars Crimson seedless and Sugraone were compared to nontransformed wild type plants regenerated from the same calli. The mutants grown and maintained in greenhouse conditions for over one year did not show any evident growth phenotype, and were not distinguishable from non-edited plants (**Figure 2**).

**Figure 3:**
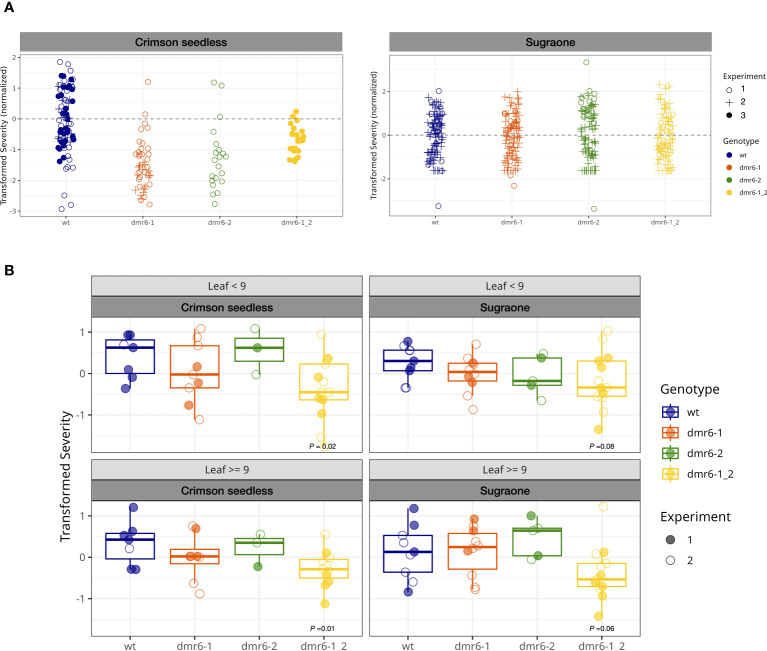
**(A)** DM-assay on detached leaf-discs of young plants. Bullets represent the relative severity (logit transformed) of leaf discs at 7 dpi in Crimson seedless and Sugraone collected in three experiments. **(B)** *In planta* DM-assay. Boxplots show average severity (logit transformed, colored bullets) of individual greenhouse-adapted plants collected from two inoculation experiments per cultivar. Each experiment included multiple lines per genotype for a total of 85 individual plants, as described in the methods. *P* values of significant differences are indicated.

A preliminary artificial inoculation with *P. viticola* was performed on detached leaf-discs of young plants (up to 10 leaves on the main shoot, **Figure 3A**). This experiment suggested that single and double mutants were less susceptible than the wild type in Crimson seedless, while no relevant differences between genotypes was observed in Sugraone (**Figure 3A** and **Figure S2**). As expected, we observed a reduction of susceptibility with increasing leaf age (**Figure S2**).

To evaluate the effect of mutations in *DMR6* genes on DM-resistance with the support of a robust statistical method, we performed two independent *in planta* inoculation assays on a large number of greenhouse-acclimatized plants (85) including multiple independent lines in both cultivars (**Figures 3B**, **S3**), and data were statistically analyzed with a Generalized Linear Model. Plants tested in these experiments were older than those used in the detached leaf-discs assay, and counted up to 30 leaves on the main shoot. The average severity (logit transformed) of each individual plant was calculated for young leaves (*<*9: 3*^rd^* to 9*^th^* leaf) and older leaves (≥9), based on their position on actively growing shoots; the data are summarized in boxplots of **Figure 3B** (grouped by genotype) and **Figure S3** (grouped by line). According to the statistical model, only the double mutant *dmr6-1_2* always showed a significant reduction in susceptibility to as compared to wild type plants in both cultivars, while DM knock-out mutations in either *VviDMR6-1* or *VviDMR6-2* were ineffective in obtaining any level of reduced susceptibility (**Figure 3B**). The reduced susceptibility observed in the double mutant is consistent across experiments, despite the high variability observed–due to different cultivars, lines within each genotype, and plants within each line– especially in older leaves, with *P* values of 0.01 for Crimson seedless and 0.06 for Sugraone.

Consistently with the experiment on detached leaf-discs performed on young plants, reduced susceptibility of the double *dmr6-1_2* is more evident in Crimson seedless than in Sugraone (*P* values of 0.02 and 0.08, respectively). This suggests that the effectiveness of the simultaneous knock out of *VviDMR6-1* and *VviDMR6-2* may depend on the genetic background, and will require specific assessment for each cultivar.

### The *dmr6-1_2* double mutant accumulates higher levels of SA

**Figure 4:**
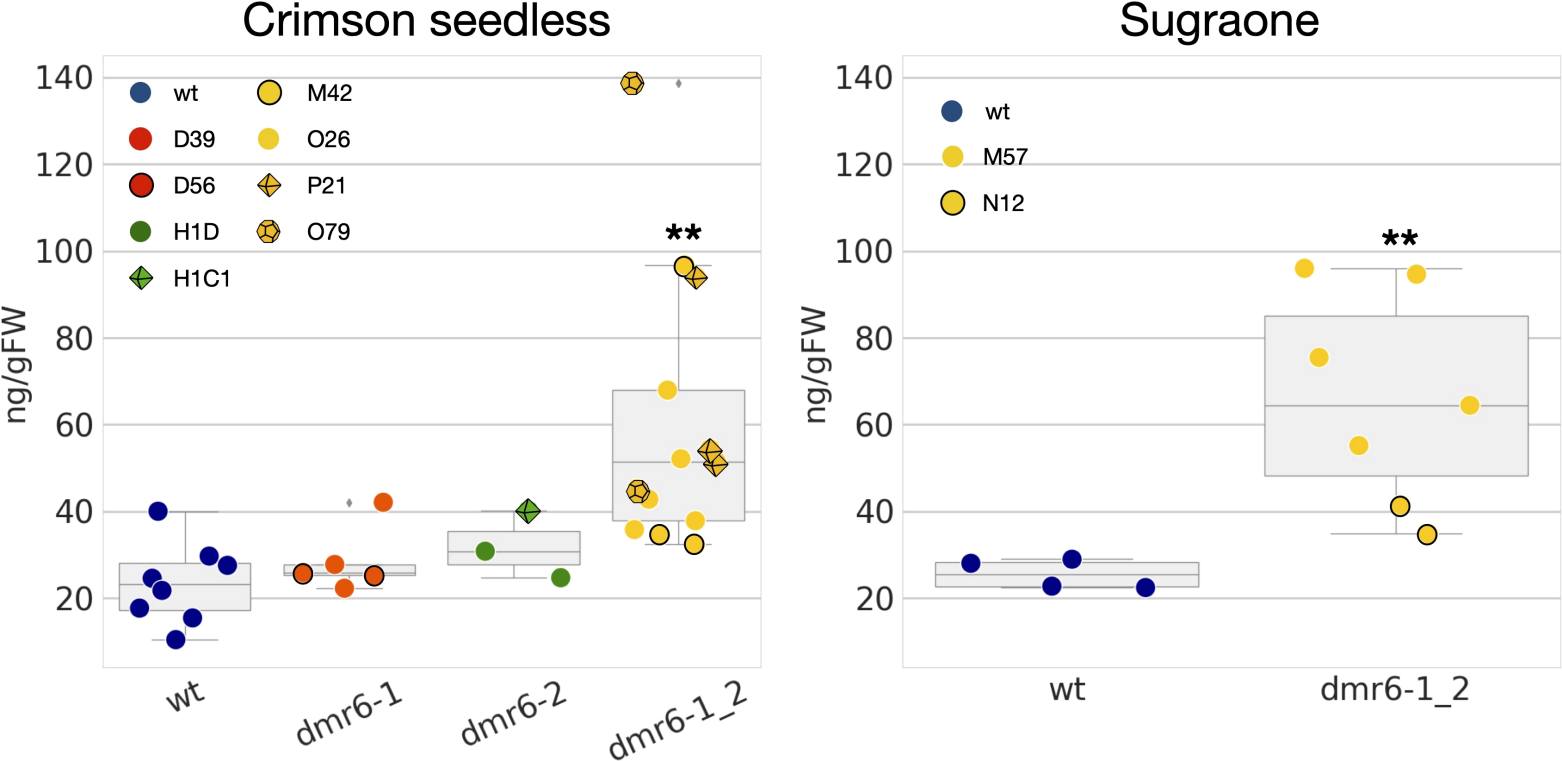
Quantification of free SA in the two cultivars. Data points represent the SA content in different biological replicates (10^th^-11^th^ leaf of individual plants) of wt (blue), *dmr6-1* (red), *dmr6-2* (green) and *dmr6-1_2* (yellow) mutants. Different lines within each genotype were analyzed as described in the legend: D39, D56 (*dmr6-1*), H1D, H1C1 (*dmr6-2*), O26, P21, O79, M42 (double mutants, Crimson seedless), and M57 and N12 (double mutants, Sugraone). Significantly different groups are indicated by asterisks (**P<0.01).

Previous results in Arabidopsis and tomato (Zeilmaker et al., 2015; de Toledo Thomazella et al., 2021) suggest that improved DM-resistance of *dmr6* mutants is due to accumulation of endogenous SA. We therefore measured the content of free SA in leaves of unchallenged plants, and our results indicate indeed a higher accumulation of SA in the double mutant than in the wild type in both cultivars (**Figure 4**). However, higher levels of SA were not observed in *dmr6-1* or *dmr6-2* single mutants (Crimson seedless), indicating that elevated SA is only obtained by the double knock out of both *DMR6* genes, at least in plants unchallenged by pathogens. This may be due to a partly redundant function of *VviDMR6-1*, *VviDMR6-2* and the three grapevine *DLO* genes in maintaining SA homeostasis, which will need further investigation.

### Concluding remarks

Only the simultaneous knock-out of *VviDMR6-1* and *VviDMR6-2* is effective in reducing susceptibility to DM–as compared to non-edited plants–and is required to increase the level of endogenous SA, while mutations in either of the individual genes seem ineffective. We therefore conclude that editing of both genes may be required to effectively control DM in the field, implying a possible increase in the chances of unwanted phenotypes. Consequently, additional research including tests in experimental vineyards are needed to better understand *DMR6*-based resistance. The need for dual mutations to successfully achieve DM-control poses a challenge in obtaining DM-resistant plants through gene editing using the existing DNA-free technology.

## Data availability statement

The datasets presented in this study can be found in online repositories. The names of the repository/repositories and accession number(s) can be found below: https://www.ncbi.nlm.nih.gov/genbank/, PRJNA909177.

## Author contributions

LG, JV, TZ, and CM conceived the experiments; TZ cloned the vectors used for transformations and performed NGS sequencing; OG and LG scored severity in the DM-assays on plants; SS and US produced the non transgenic *dmr6-2* edited lines from protoplasts; PF performed statistical analysis; DM and UV performed Mass Spectrometry analyses. LG produced and maintained grapevine calli, produced, screened and maintained transgenic plants, performed the phenotypic analyses (DM assays, SA measurements), analyzed the results, and wrote the manuscript. All the authors reviewed and approved the manuscript.
