# Supplementary material for: Simultaneous editing of two DMR6 genes in grapevine results in reduced susceptibility to downy mildew
Source: Front Plant Sci. 2023 Aug 21;14:1242240. doi: 10.3389/fpls.2023.1242240 (PMC10486898; doi:10.3389/fpls.2023.1242240)
Supplement: Supplementary file 1 [file DataSheet_1.pdf]

Supplementary Material

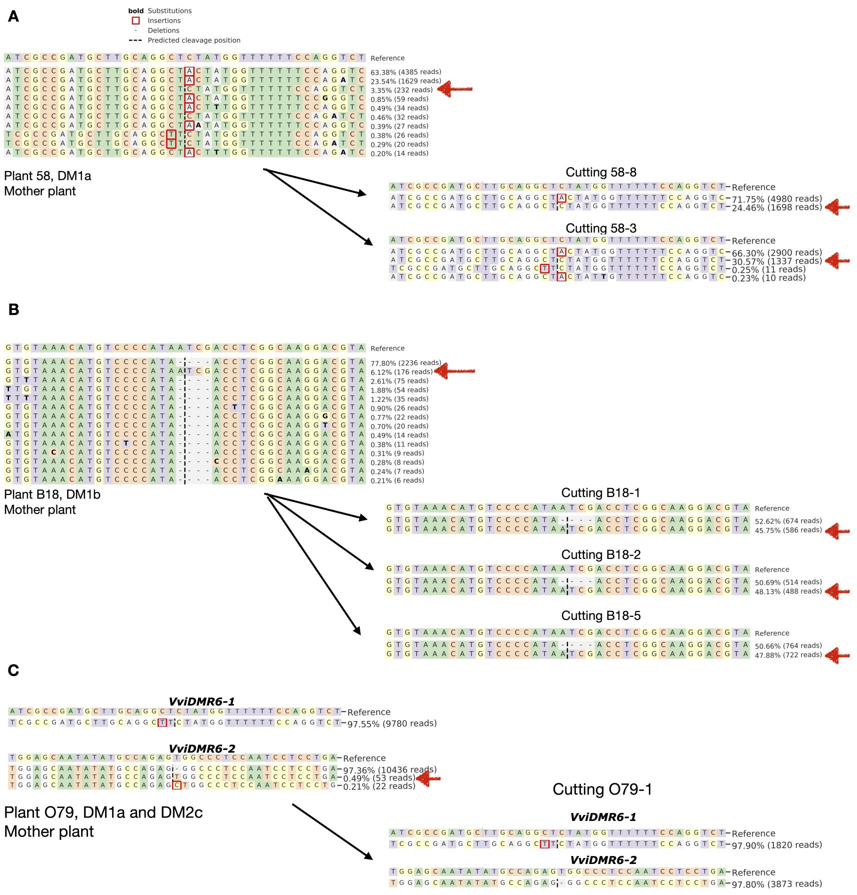

Figure S1. Genetic instability of lines with editing 90-99% in propagated cuttings
The figures show the CRISPResso output of NGS (next generation sequencing) of the DNA sampled from leaves of a mother plant (the plant regenerated from callus), and cuttings propagated over time from such plant. Non-edited sequences identical to the reference one are indicated by a red arrow. Plant 58 showed 3.4% of non-edited DNA in the mother plant, and a much larger portion in its cuttings **(A)**. The same applies for plant B18: the mother plant shows 6.1% of non-edited reads, and the cuttings are heterozygous **(B)**. In the completely-edited plant line O79, the mother plant shows <1% of non-edited reads, and the genotype was stable over time in its cuttings **(C)**.

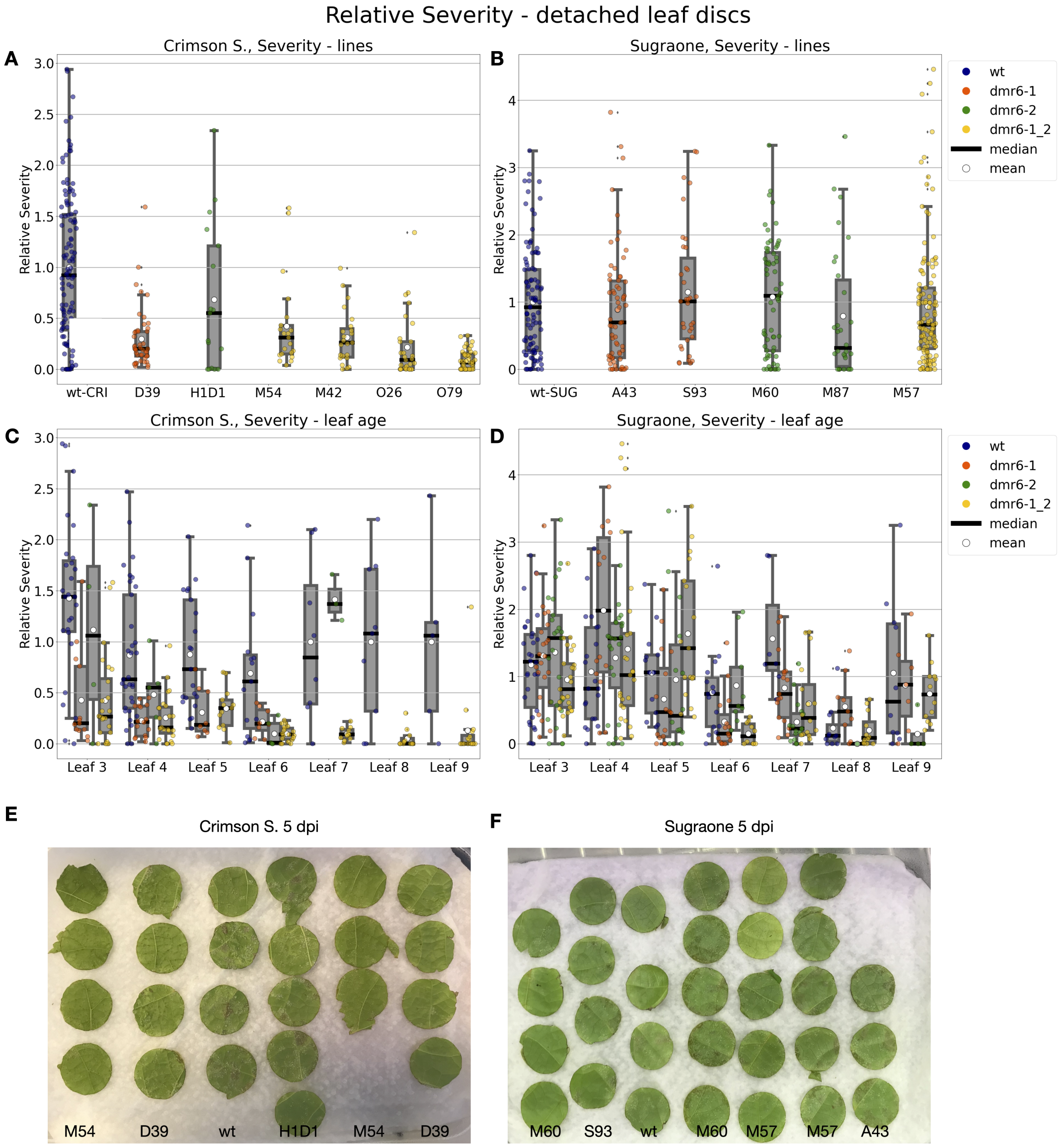

Figure S2. Detached leaf-discs assay
Colored bullets represent the relative severity of individual leaf discs at 7 dpi. Data are normalized to the mean severity of the wild type within each experiment. Boxplots indicate the dispersion of data in different lines **(A)** and leaves **(C)** of Crimson seedless and in different lines **(B)** and leaves **(D)** of Sugraone plants. Leaf age **(C, D)** was inferred by the leaf position on a growing shoot. Representative images of leaf discs of different lines with sporulation at 5 dpi are shown for Crimson seedless **(E)** and Sugraone **(F)**. Leaf discs of different lines are organised in different columns, and their identity is indicated at the bottom of each column.

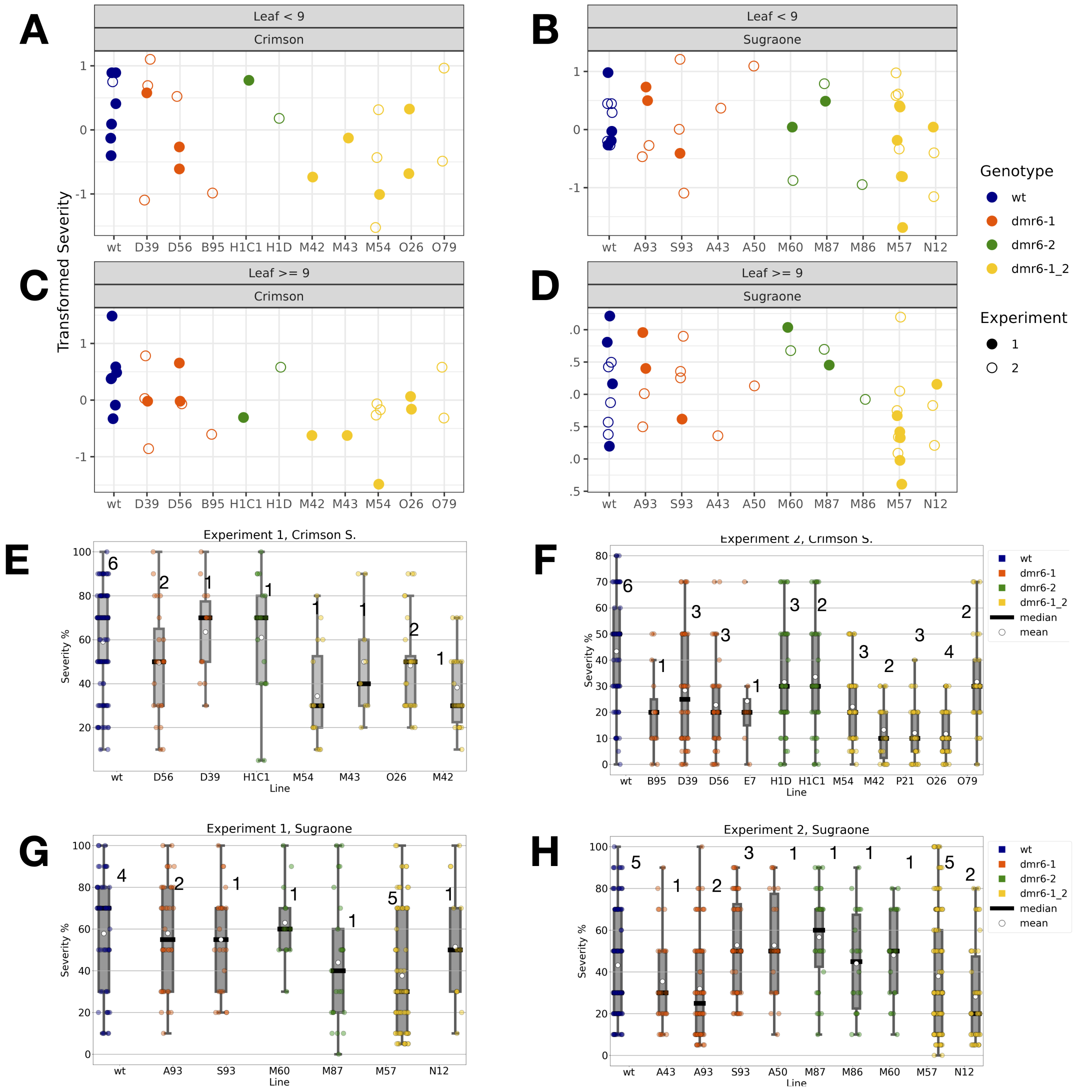

Figure S3. *In planta* DM-assay: severity in different lines
The plots in this figure are additional representation of data summarized in figure 3 B to show variability among lines.
(A-D) Colored bullets represent the transformed (logit) severity of younger **(A, B)** and older leaves **(C, D)** of individual plants at 6 dpi in the different lines of Crimson seedless **(A, C)** and Sugraone **(B, D)**. Leaf age is inferred by leaf position on a growing shoot: younger leaves are from the 3rd to the 8th on a growing shoot, and older leaves are from the 9th up to the 27th. Filled circles are relative to experiment 1 and empty circles are relative to experiment 2.
(E-H) Colored bullets in the boxplots represent the leaf severity (%) of individual leaves of Crimson seedless **(E, G)**, and Sugraone **(F, H)** collected from the two inoculation experiments: experiment 1 **(E, F)** and experiment 2 **(G, H)**. Each experiment included multiple lines per genotype and multiple plants per line (indicated by the numbers in plots), as described in the methods.

Table S1. Transgenic grapevine plants regenerated from transformed calli and sequenced.

Plants sequenced and edited in *VviDMR6-1* sgRNA DM1a

Plasmid	Cultivar	Sequenced	WT	Edited	Edited >99%	Edited 90-99%	Edited 1-90%
pDM1a2a	Chardonnay	23	15	8	0	0	8
pDM1a2a	Sugraone	169	25	144	5	2	137
pDM1a	Crimson S.	71	28	43	3	1	39
pDM1a	Microvine	1	0	1	0	0	1
pDM1a	Sugraone	164	55	109	30	8	71
pDM1a2c	Crimson S.	87	25	62	5	0	57
pDM1a2c	Sugraone	26	9	17	5	0	12
	Total	541	157	384	48	11	325

Plants sequenced and edited in *VviDMR6-1* sgRNA DM1b

Plasmid	Cultivar	Sequenced	WT	Edited	Edited >99%	Edited 90-99%	Edited 1-90%
pDM1b	Sugraone	100	9	91	4	8	79
	Total	100	9	91	4	8	79

Plants sequenced and edited in *VviDMR6-2* sgRNA DM2a

Plasmid	Cultivar	Sequenced	WT	Edited	Edited >99%	Edited 90-99%	Edited 1-90%
pDM1a2a	Chardonnay	21	21	0	0	0	0
pDM1a2a	Sugraone	143	143	0	0	0	0
pDM2a	Sugraone	2	1	1	0	0	1
pDM2a	Crimson S.	92	81	11	0	0	11
pDM2a	Merlot	1	0	1	0	0	1
pDM2a	Thompson S.	52	11	41	1	0	40
	Total	311	257	54	1	0	53

Plants sequenced and edited in *VviDMR6-2* sgRNA DM2b

Plasmid	Cultivar	Sequenced	WT	Edited	Edited >99%	Edited 90-99%	Edited 1-90%
pDM2b	Crimson S.	65	63	2	0	0	2
pDM2b	Sugraone	133	128	5	0	0	5
	Total	198	191	7	0	0	7

Plants sequenced and edited in *VviDMR6-2* sgRNA DM2c

Plasmid	Cultivar	Sequenced	WT	Edited	Edited >99%	Edited 90-99%	Edited 1-90%
pDM1a2c	Crimson S.	92	4	88	78	2	8
pDM1a2c	Sugraone	29	1	28	25	0	3
	Total	121	5	116	103	2	11

Table S2. Compound-specific instrumental parameters used in the analytical method- Mass spectrometry analysis.
DP: declustering potential, EP: entrance potential, CE: collision energy, CEP: collision energy potential.

Name	Ionization Mode	Precursor Ion	Q1 Product Ion	DP	EP	CE	Collision CEP
2,5-DHBA	[M – H]-	153	109	-80	-12	-18	-10
2,3-DHBA	[M – H]-	153	109	-80	-12	-20	-12
SA	[M – H]-	137	93	-80	-10	-25	-10
SAD4	[M - H]-	141	97	-100	-10	-24	-10

Name	Q2 Product Ion	DP	EP	CE	Collision CEP	tr
2,5-DHBA	81.1	-80	-12	-24	-10	7
2,3-DHBA	91	-80	-12	-35	-12	8.3
SA	65	-80	-10	-38	-10	11.4
SAD4	69	-100	-10	-40	-10	11.3
